# Effects of Two Different Dietary Calcium Concentrations on Bone Density and Skin Microbiome in Lemur Tree Frogs (*Agalychnis lemur*)

**DOI:** 10.3390/ani16040660

**Published:** 2026-02-19

**Authors:** M. Graciela Aguilar, John Tuminello, Ashleigh Godke, Ariana Tashakkori, Aspen Settle, Haerin Rhim, Lillian Dickson, Kenneth L. Matthews, Mark Yacoub, Kaylie Zapanta, Janina A. Krumbeck, Mark A. Mitchell

**Affiliations:** 1 Department of Veterinary Clinical Sciences, School of Veterinary Medicine, Louisiana State University, Skip Bertman Drive, Baton Rouge, LA 70803, USA; magui32@lsu.edu (M.G.A.);; 2 Department of Physics & Astronomy, Louisiana State University, Baton Rouge, LA 70803, USAkipmatth@lsu.edu (K.L.M.II); 3 MiDOG Animal Diagnostics LLC, 14762 Bentley Circle, Tustin, CA 92780, USA; myacoub@midogtest.com (M.Y.);; 4 Department of Pathobiological Sciences, School of Veterinary Medicine, Louisiana State University, Skip Bertman Drive, Baton Rouge, LA 70803, USA

**Keywords:** *Agalychnis lemur*, amphibian, anuran, frog, ex situ, nutrition, Ca:P ratio, micro-CT scan, bone density

## Abstract

The lemur tree frog (*Agalychnis lemur*), a critically endangered species, can benefit from ex situ conservation programs; however, managing amphibians under human care presents challenges, including the provision of appropriate nutrition. House crickets (*Acheta domesticus*), a common feeder insect, have an inverse calcium to phosphorus ratio (Ca:P; 0.15:1) and low calcium content (<0.3%). While gut-loading crickets with an 8% calcium diet can improve their calcium concentrations, no study has assessed the effects of dietary calcium on bone development in *Agalychnis* spp. Moreover, no study has examined how diet impacts the gut–skin axis and skin microbiome of these frogs. This study examined how crickets gut-loaded with either a 1.3% or 8% calcium diet affected lemur tree frog bone density and skin microbiome. We hypothesized that frogs consuming the 8% calcium diet would exhibit significantly higher Hounsfield units (HU; bone density) over time, as measured by micro-computed tomography (mCT), and that dietary calcium concentration would have no effect on skin bacterial and fungi microbiomes. Eleven juvenile lemur tree frogs underwent mCT scans at baseline and 90 and 180 days. Total body volume of interest analysis showed a significant increase in HU in the 8% calcium group compared to the 1.3% group (F = 9.9, *p* = 0.01). There was no significant difference noted in the alpha or beta diversities for the bacterial and fungal microbiomes between dietary groups. This study provides the first evidence of dietary calcium’s impact on bone density in lemur tree frogs, offering valuable insights for improving ex situ management of this species.

## Simple Summary

Threatened species, such as lemur tree frogs (*Agalychnis lemur*), benefit from conservation programs. However, maintaining amphibians under human care presents challenges, including providing appropriate nutrition. Many frogs under human care are fed crickets, but these insects are naturally low in calcium. This study examined whether gut-loading crickets with a high-calcium diet could improve lemur tree frog bone density and affect bacterial and fungal skin communities (microbiome). There was no significant difference in the skin microbiome between groups; however, frogs offered high-calcium crickets had significantly higher bone density compared to frogs offered low-calcium crickets. These findings emphasize the importance of proper nutrition in protecting endangered frogs in captivity. Enhancing dietary calcium can help zoos and conservation programs raise healthier frogs, increasing their chances of survival and successful breeding. This research provides valuable insights for global amphibian conservation efforts by ensuring frogs under human care receive the necessary nutrition to thrive.

## 1. Introduction

Amphibians are the most threatened vertebrate group, with 41% of their nearly 9000 species threatened with extinction [1,2]. This global crisis is driven by anthropogenic pressures, including habitat loss, climate change, emerging infectious diseases, fire, pollution, invasive species, and overexploitation [3,4]. Tropical and subtropical amphibian populations are particularly vulnerable to these threats [3,5,6]. The lemur tree frog (*Agalychnis lemur*), native to Costa Rica, Panamá, and Colombia, exemplifies this crisis, having experienced a population decline of 80–95% over the past two decades [7]. In response, the 2007 Amphibian Conservation Action Plan advocated for the establishment and management of ex situ survival assurance colonies as a short-term solution to safeguard threatened species, while in situ conservation efforts address these causes in the medium and long term, offering additional time for conservation efforts and eventual reintroductions [8,9]. However, raising amphibians in ex situ settings is challenging. Amphibians represent one of the most understudied vertebrate groups, resulting in gaps in knowledge about even basic aspects of husbandry and nutrition [10]. As a result, it is common to observe clinical manifestations of nutritional disease in captive amphibians [11].

Nutritional secondary hyperparathyroidism (NSHP) is one of the most common diseases reported in captive insectivorous animals, including amphibians [10,12,13,14]. This type of metabolic bone disease manifests in animals as weakness, tetany, poorly mineralized bones, and pathological fractures and is often the result of calcium-deficient insect-based diets [10,15]. Most commercially available insects are naturally deficient in calcium, with calcium-to-phosphorus ratios (Ca:P) that are inverse to the recommended 1:1 ratio required for healthy bone development for vertebrates [16,17]. For example, the domestic house cricket (*Acheta domesticus*) has <0.3% calcium on a dry matter basis (DM), resulting in a Ca:P ratio of only 0.15:1 [12,18,19,20].

Several methods for increasing calcium concentrations in insects have been explored, with dusting insects with calcium supplements and gut-loading them with a high-calcium diet being the most widely used [12,21]. While dusting has proven ineffective for retaining sufficient calcium on insects, the gut-loading technique has been found to be more consistent [18,21,22,23,24]. Aguilar et al. [25] recently refined a practical and effective method of gut-loading crickets in 6 h using an 8% high-calcium gut-loading diet; however, the impact of this and other methods remains largely unexplored with respect to amphibian health and needs to be further evaluated. It is important not to assume that all species of amphibians will digest and assimilate nutrients (e.g., calcium) in a similar fashion; otherwise, it would be analogous to thinking that rats, dogs, and humans similarly digest and assimilate calcium, which they do not [26].

In addition to the skeletal system, amphibian skin has been recognized as an important site for calcium storage, with up to 30% of total body calcium storage in some anuran species [27]. Calcium plays a critical role in skin physiology by regulating skin functions, including keratinocyte differentiation, skin barrier formation, and permeability barrier homeostasis [28]; it also plays fundamental roles in modulating mucus signaling within the skin, which can influence the developmental transition of the chytrid fungus (*Batrachochytrium dendrobatidis*; Bd), the most significant pathogen contributing to global amphibian declines [29,30]. The skin microbial communities, members of the skin microbiome, support immune function and maintain skin integrity, acting as a primary defense against pathogens, including Bd, but can also be influenced by multiple factors, including nutrients [31,32,33]. The effect of dietary calcium on the skin microbiome should also be considered when assessing their nutritional health; however, no study has examined whether dietary calcium concentrations affect the amphibian skin microbiome.

The purpose of this prospective experimental study was to determine the impact of gut-loading domestic house crickets with two different calcium concentrations (1.3% vs. 8%) on the growth, bone density, and skin microbiomes of subadult lemur tree frogs. The main objective was to measure the total mean Hounsfield units (HU) of the whole body of the lemur tree frogs using micro-computed tomography (mCT) scans at baseline, 90 days, and 180 days after being fed gut-loaded domestic house crickets and offered two different gut-loading diets. The second objective was to determine whether the skin microbiomes differed between frogs offered the two different cricket diets. The hypotheses being tested were as follows: (1) there would be no difference in growth rate between the two groups; (2) there would be a significant difference in the HU values over time for both groups; (3) the frogs fed the crickets gut-loaded with 8% calcium diet would have significantly higher total body HU values (total volume of interest, TVOI) than the frogs offered the crickets fed the 1.3% calcium diet; (4) there would not be differences in HU measures between select cross-sections (region of interest, ROI) and whole bones (bone volume of interest, BVOI); and (5) there would not be a significant difference in the skin microbiomes between diet groups.

## 2. Materials and Methods

### 2.1. Animals and Husbandry

The research was done in accordance with the rules and regulations set forth by the Louisiana State University’s Animal Care and Use Committee (protocol #22-094).

Eleven unsexed, captive-bred, approximately 4.5-month-post-metamorphic lemur tree frogs were obtained from a commercial breeder (Smart Exotics, Falling Waters, WV, USA). The animals were housed in individual vertical 42 L (11-gallon) glass cages (Tropical Vertical Kit; Zilla Products, Franklin, WI, USA). Each enclosure had natural plants (*Epipremnum aureum*), a large water bowl, and a paper towel substrate. The substrates were exchanged weekly, and cages were spot-cleaned daily. The room temperature and humidity were maintained at 22.22–25 °C (72–77 °F) and 70–99%, respectively. Space heaters (Dayton Portable Electric Heater, Lake Forest, IL, USA) and humidifiers (Greenland 5.3Gal Commercial Humidifier for Large Rooms, Turbo, Las Vegas, NV, USA) were used to maintain these parameters. The husbandry was intended to mimic the natural conditions of the lemur tree frog through controlled temperature and humidity ranges and the presence of live vegetation. Non-ultraviolet B fluorescent lighting was used to provide a 12 h photoperiod. Dechlorinated tap water was used to fill the water bowls, mist the cages twice daily, and fill the room humidifiers. Prior to starting the trial, the frogs were examined and found to be healthy with no clinical signs of disease. Three serial negative fecal endoparasite examinations were also performed to confirm the health of the frogs. The staff handling the frogs always wore nitrile gloves that were sprayed with dechlorinated water for all frog-handling procedures. The animals were acclimated to these settings for four months (between December and March), during which time they were offered five 2-week-old house crickets *(Acheta domesticus*) (Fluker Farms, Inc., Port Allen, LA, USA) daily that were gut-loaded with a cricket base diet with 1.3% calcium (DM) (Fluker Farms, Inc.).

### 2.2. Diets

Two different calcium concentrations were tested in the cricket diets. The control diet consisted of a cornmeal cricket-based diet (Fluker Farms, Inc.) with 1.3% calcium DM (1.28 ± 0.26%, min–max: 1.26–1.31%). The control diet was modified to create a treatment diet (8% calcium DM) by adding 200 g/kg of calcium carbonate (Calcium without Vitamin D_3_ supplement, Fluker Farms, Inc.), following the methodologies described by Aguilar et al. [25] and Finke [22]. The control and treatment diets were both supplemented with vitamin A (10,000 µg/kg) and 30 mL/kg corn oil, thoroughly mixed with a blender (2 Speed Hand Blender 59762FG, Hamilton Beach Brands, Inc, Glen Allen, VA, USA) until homogeneous, and then heated to 76.7 °C (170 °F) for 30 min. The diets were stored in a refrigerator at 4 °C (39.2 °F) until being used for the trial. During the experiment, the frogs were offered 2-week-old house crickets weighing 30–50 mg (Fluker Farms, Inc.). Crickets were gut-loaded with the respective diets for six hours prior to being offered to the frogs, following the protocol outlined in Aguilar et al. [25].

### 2.3. Feeding Trial

This study was conducted between April and October 2023. The frogs were randomly divided into two groups using a random number generator (random.org). The control group (*n* = 5) was offered crickets gut-loaded on the 1.3% calcium DM diet, whereas the treatment group (*n* = 6) was offered crickets fed the 8% calcium DM diet. Each study group (control and treatment) was housed on different sides of the room and had their own specific transparent, plastic rectangular communal bins to house their crickets (Sterilite storage tote, 32 L [8 gallon]; Sterilite Corp., Townsend, MA, USA) and transparent plastic round cup (Josh’s Frogs 32oz insect cup and vented lid; Josh’s Frogs, Owosso, MI, USA) to gut-load the crickets. Every morning (first day), crickets that were to be gut-loaded and offered to the frogs were collected from their communal bins, where they were offered the control diet and transferred to a smaller container to induce a 24 h fasting period. The following morning (the second day), the fasted crickets were transferred to a second container with 50 g of their respective control or treatment diet. The crickets were gut-loaded for 6 h (9 a.m. to 3 p.m.). Moisture was not provided during the fasting or gut-loading process to maximize their ingestion of the respective diets [25]. Because the frogs are nocturnal, they were offered five crickets in a feeding station (16 oz. white plastic cup, Dollar Tree, Inc., Chesapeake, VA, USA) once the room light was turned off (4 p.m.). On the following morning (9 a.m.), uneaten crickets were counted and removed from the enclosures. The frogs were fed every day for 180 days, and they were examined and weighed weekly. Samples of gut-loaded crickets from each group were also randomly collected for testing over the study period.

### 2.4. Nutritional Analysis

Samples of diets and gut-loaded crickets were analyzed for calcium, phosphorus, DM, and moisture content on a DM basis. The feed and cricket samples were collected weekly, but only a subset of samples was randomly collected for testing over the study period. A random number generator (random.org) was used to select the samples for testing. All analyses were performed by Zooquarius Laboratory Services (Dairy One Laboratory, Ithaca, NY, USA). Samples (15–20 g) were stored in a −80 °C (−112 °F) freezer before being transported to the laboratory on frozen gel packs. Prior to chemical analysis, diets and crickets were first homogenized and dried in a forced air oven at 60 °C (140 °F) for at least four hours to obtain DM content (and thus respective moisture content) using NFTA Method 2.2.1.1 [34]. Samples were then placed in a drying oven at 135 °C (275 °F) for 2 h following AOAC Official Method 930.15 [35]. For the minerals, the diets and crickets were pre-digested using HNO_3_, HCl, and H_2_O_2_ and further digested using a CEM Microwave Accelerated Reaction System (MARS6) (CEM Corporation, Matthews, NC, USA). All mineral concentrations were then analyzed using a Thermo iCAP Pro XP Inductively Coupled Plasma Radial Spectrometer (Thermo Scientific, Waltham, MA, USA) [36].

### 2.5. Bone Density Analysis

**Figure 1:**
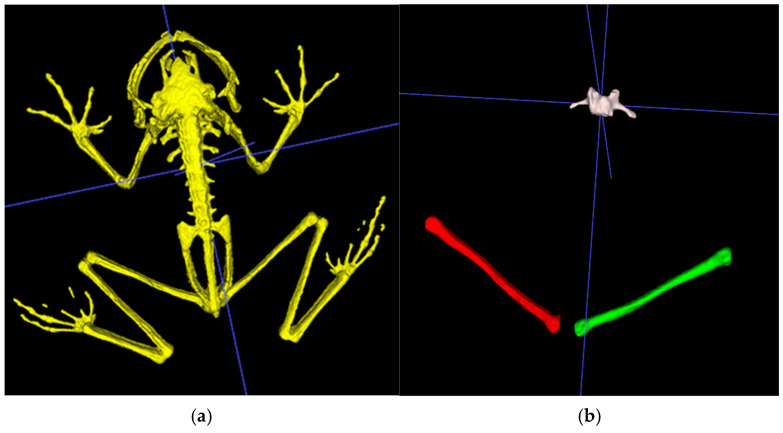
Examples of three-dimensional volume segmentations of a lemur tree frog using ITK-SNAP software: (**a**) total body bone density analysis; (**b**) bone volume of interest for left (red) and right (green) femurs and the third vertebra (white). Blue lines represent the 3D cursor selection.

**Figure 2:**
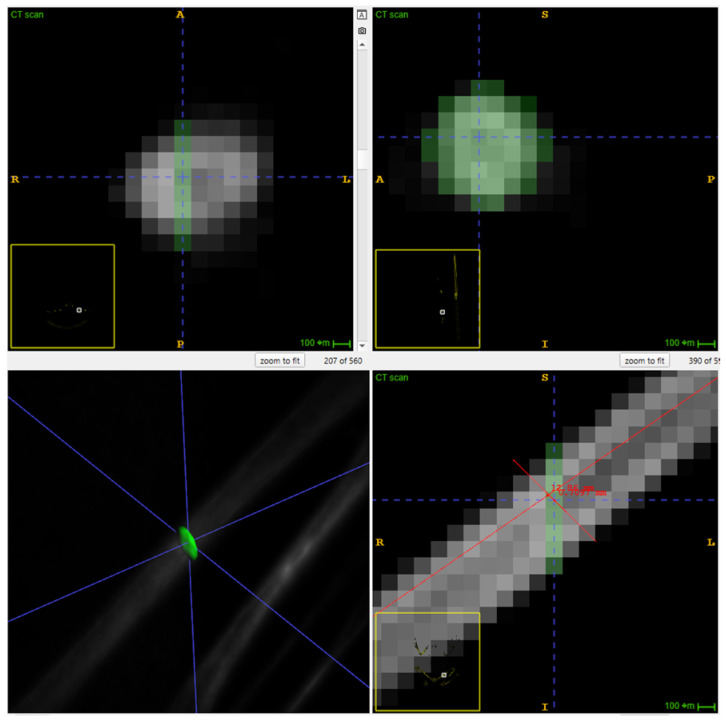
Cross-sectional region of interest of a right femur from a lemur tree frog using ITK-SNAP software. Blue lines represent the 3D cursor selection; the measurements represent the mid-diaphysis location used for the ROI.

**Figure 3:**
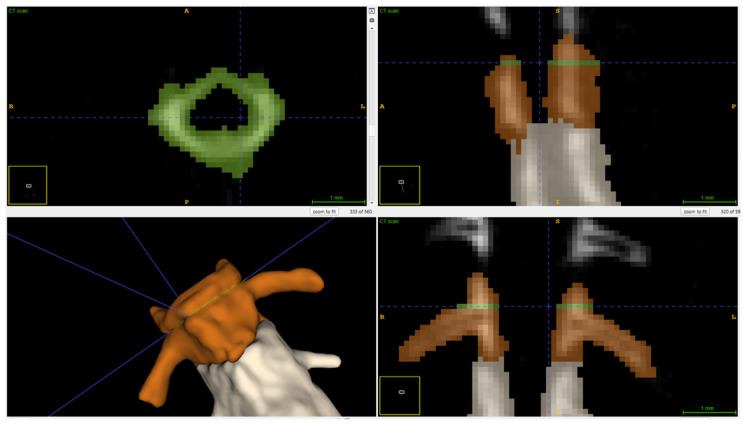
Cross-sectional region of interest of the third vertebra from a lemur tree frog using ITK-SNAP software. Blue lines represent the 3D cursor selection.

Frog bone density was measured using selected ROI, BVOI, and TVOI, obtained using a single gantry rotation mCT scanner (Triumph^®^ II, Preclinical CT, Trifoil Imaging, Northridge, CA, USA). The mCT scans were collected before starting the feeding trial (baseline) and at 90 and 180 days after starting the trial. To minimize movement artifacts, the frogs were sedated using 800 mg/L of tricaine methanesulfonate (Syncaine; Syndel, Inc., Ferndale, WA, USA) [37]. The frogs were positioned in ventral recumbency in a transparent plastic box (Small clear plastic storage container box 35 × 35 × 18 mm, Easytle^®^, Amazon, Seattle, WA, USA). The mCT was performed using an X-ray energy of 75 kV, an exposure time per projection of 230 ms, a tube current of 110 mA with a product of 25 mAs, a reconstructed voxel isotropic size of 0.154 × 0.154 × 0.154 mm^3^ and a reconstructed field view of 91 × 91 × 86 mm, and a radiation dose of <200 mGy per scan. The bone density measurements were obtained using the total mean HU, which was calculated with ITK-SNAP software (4.0.2; University of Pennsylvania, Philadelphia, PA, USA). The TVOI was measured for each mCT (days 0, 90, and 180). The skeleton was separated from the soft tissue using the three-dimensional segmentation tool. The setting included a lower threshold of 7000 HU, no maximum HU constraints, a smoothing factor of 0.2, and a bubble radius of 0.5. The intuitive mode was applied with a region competition force of 1 and a smoothing force of 0.2, running up to 9999 iterations (Figure 1a). For the third mCT scan (180 days), BVOI and ROI were measured. For the specific BVOI, both femurs (left and right) and the third vertebra were selected. The selections were performed using the same settings described for the TVOI (Figure 1b). The femurs were also used for the cross-sectional ROI; however, a minimum contrast adjustment of 7000 was applied. For the femoral ROI, bone length was measured, and the sample was collected at the mid-diaphysis in the sagittal view as an oblique ROI using the paintbrush inspector with the round brush shape (Figure 2). For the vertebral ROI, the same settings were applied; however, the ROI selection was made cranial to the transverse process (Figure 3).

### 2.6. Skin Microbiome

At the end of the trial, whole-body skin swabs were collected from each frog using the small-sized swab collection kit from MiDOG (MiDOG animal diagnostics LLC, Tustin, CA, USA). One handler restrained the frog with sterile latex-free gloves (SensiCare PI Surgical Gloves, Medline Industries LP, Northfield, IL, USA), while a second person, wearing nitrile gloves (FitGuard Touch Nitrile Exam Gloves, Medline Industries LP, Northfield, IL, USA), swabbed the dorsum and ventrum (10 rotations each with moderate pressure) with the sterile collection swab (Zymo Research Corp., Irvine, CA, USA). No pre-cleaning was performed. Each swab was placed into a sterile sample collection tube with a DNA/RNA preservative (DNA/RNA Shield; Zymo Research Corp.) and shaken 10 times. Samples from 10 of the frogs were collected and stored at ambient temperature until submission for next-generation sequencing analysis (NGSA) at MiDOG animal diagnostics LLC. Previously obtained samples processed with the same methods were used for pre-trial comparison. These samples were collected (February 2023). The NGSA targeted the bacterial microbiota using the 16S rRNA V1–V3 region for bacteriome analysis and ITS2 for mycobiota analysis [38].

### 2.7. Statistical Analysis

Calcium, phosphorus, total DM, and moisture contents of feed and gut-loaded crickets were analyzed as percentages based on DM content. Continuous data (HU, body weight, food intake, calcium, phosphorus, DM, and moisture content) were evaluated for normality using the Shapiro–Wilk test, skewness, kurtosis, Q–Q plots, and residuals. Normally distributed data are reported as mean ± standard deviation (SD) and minimum–maximum (min–max) values; non-normally distributed data are reported as median, 10–90 percentiles (%), and min–max.

Independent sample *t*-tests were used to analyze calcium, phosphorus, Ca:P ratios, moisture, and DM between diets (1.3% and 8%) and for gut-loaded cricket samples. Levene’s test was used to assess homogeneity of variance. One-tailed hypotheses were used for the calcium and Ca:P ratio data; two-tailed hypotheses were used for the other dependent variables. Because phosphorus did not meet the assumption of normality, a Mann–Whitney U test was used.

The baseline frog weight, total crickets eaten, and average daily cricket intake were compared between the diet groups using Levene’s test for homogeneity and independent-sample *t*-tests with non-directional hypotheses. Pearson’s correlation assessed the relationship between total crickets consumed and percent weight gain.

A linear mixed model was used to determine whether body weight was affected by group, time, or their interaction. Group and time were included in the model as fixed factors, while frog served as the random effect. Bonferroni corrections were applied to post hoc comparisons. Akaike’s information criterion was used to determine the best model fit. The same statistical model was used to evaluate the effect of time (baseline, 90 days, and 180 days), group (control vs. treatment), and their interaction on TVOI HU.

Sex and weight were not included in the mixed models. Sex was excluded because there was only a single female and a single male in the control and treatment groups, respectively, and inclusion of the variable led to a type II error for the model. While the inclusion of weight in the model was used to examine bone density, its inclusion as a covariate led to model failure because of the number of predictor variables and the small sample size. Therefore, both variables were evaluated separately. The previous paragraph addressed body weight, while sex was evaluated using an independent-sample *t*-test for the TVOI HU data, regardless of time or group, as well as for daily cricket consumption, initial weight, and final weight.

Independent-sample *t*-tests were also used to determine differences by group for the final mCT scans, BVOIs, and ROIs. Levene’s test was used to test for homogeneity of variance.

For the microbiome analysis, alpha diversity metrics (observed species, Shannon, and Simpson) were analyzed using linear mixed models, with sex, time, and diet group and their interactions as predictors. Beta diversity was examined using a principal coordinate analysis (PCoA) with Bray–Curtis dissimilarity and PERMANOVA.

Statistical significance was set at *p* < 0.05 for all comparisons. Statistical analyses were performed using SPSS 25.0 (IBM Statistics, Armonk, NY, USA) and MicrobiomeAnalyst 2.0 (OmicsForum) [39].

## 3. Results

### 3.1. Diets

The treatment cricket diet was successfully modified, resulting in a significant increase in calcium concentrations compared with the control diet (t = −24.94, *p* < 0.001) and a significant decrease in phosphorus concentrations (t = 8.66, *p* < 0.001) (Table 1). No significant differences were observed between diets for moisture (t = 0.97, *p* = 0.36) or DM content (t = −9.21, *p* = 0.38) (Table 1). Gut-loaded crickets offered the treatment diet also showed a significant increase in calcium concentration (t = −5.7, *p* = 0.005), resulting in a significant positive Ca:P ratio (t = −5.13, *p* = 0.007) compared with the control group (Table 2). No significant differences in phosphorus (t = −0.08, *p* = 0.94), moisture (t = −0.41, *p* = 0.694), or DM content (t = 0.41, *p* = 0.694) were found between groups. (Table 2).

### 3.2. Frog Growth, Weight, and Cricket Consumption

This study was initiated when the frogs were approximately 8 months old, as subadults. The trial concluded before the animals reached 13 months old (in October), prior to reaching full adulthood. At the end of this study, the mean snout–vent length of females was 3.45 ± 0.13 cm (min–max: 3.3–3.6 cm), while males measured 2.63 ± 0.80 cm (min–max: 1.1–3.5 cm).

At the beginning of this study, the lemur tree frogs had an average weight of 2.04 ± 0.34 g (min–max: 1.59–2.77), with no significant difference between groups (t = −0.15, *p* = 0.88). At the end of this study, the animals had an average weight of 2.31 ± 0.6 g (min–max: 1.55–3.09). The final body weights were significantly different from the baseline (F = 7.54, *p* = 0.024); however, no significant difference was observed between groups (F = 1.34, *p* = 0.28), nor the interaction term group and time (F = 3.10, *p* = 0.11). The frogs exhibited a mean individual 36.14 ± 47.83% (min–max: −21.70–152) weight gain, with no significant difference between the two groups (t = −1.63, *p* = 0.14).

The frogs consumed an average of 1.74 ± 0.34 crickets (min–max: 1.28–2.28) per day, representing a daily intake of 2.5 to 4% of the frogs’ body weight. The control group consumed an average of 1.62 ± 0.38 crickets/day (min–max: 1.28–2.2), while the treatment group consumed 1.84 ± 0.31 crickets/day (min–max: 1.3–2.28); there was no significant difference between groups (t = −1.05, *p* = 0.32). The total average cricket consumption during this study was 311.45 ± 73.71 (min–max: 198–427), with no significant difference between groups (t = −0.34, *p* = 0.74). No significant correlation was found between the total number of crickets consumed and the percentage weight gain observed in the frogs over the study period (r = 0.45, *p* = 0.2).

### 3.3. Bone Density (HU)

**Figure 4:**
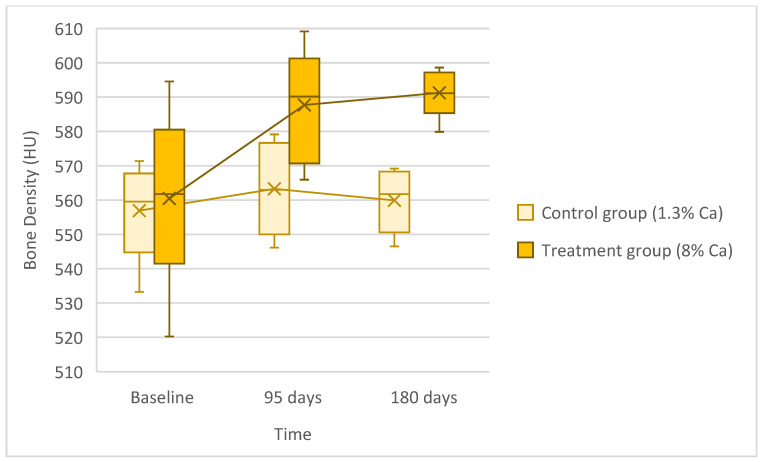
Whole-body bone density (HU) of lemur tree frogs was measured over time (baseline, 90, and 180 days) and compared between the control (1.3% Calcium diet) and treatment groups (8% Calcium diet). A significant difference was found between baseline and 180 days and between groups. Boxes: 25–75th interquartile range; X: mean; horizontal line: median; whiskers: min–max.

Whole-body TVOI mCT scans revealed significant changes in the frogs’ bone density over time (F = 4.73, *p* = 0.024) and between groups (F = 9.9, *p* = 0.013); however, no significant difference was found between the interaction of time and group (F = 2.65, *p* = 0.101). Baseline mCT scans (558.85 ± 20.09, min–max: 520.25–594.57) were lower than the 90-day mCT scans (576.60 ± 19.21, min–max: 546.15–609.17), but this difference was not statistically significant (*p* = 0.052). However, baseline HU values were significantly lower than the 180-day HU values (575.58 ± 18.30, min–max: 546.52–598.61) (*p* = 0.05). For the group, HU values for the treatment group (8% Ca diet, 579.13 ± 22.30, min–max: 520.25–609.17) were significantly higher (*p* = 0.013) than the control group (1.3% Ca diet, 560.03 ± 12.06, min–max: 533.20–579.12) (Figure 4). The BVOI and ROI mCT analyses of the right and left femurs and third vertebra were not significantly different (all *p* ≥ 0.295; Table 3). None of the frogs developed signs of NSHP over the course of this study.

Because this study was initiated with subadult frogs, they could not be sexed until the end of this study; this is also why they were not randomly allocated by sex at the beginning of this study. The control group consisted of four males and one female, while the treatment group had two males and four females. The whole bone density HU values for the females and males, regardless of group and time, were 571.91 ± 21.84 HU (min–max: 520.25–595.77) and 568.66 ± 19.59 HU (min–max: 533.20–609.17), respectively, and there was no significant difference in HU values between the sexes (t = −0.44, *p* = 0.66). Although sex did not affect HU, it did affect weight and cricket intake. No significant difference was found in the initial weight per sex at baseline (t = −2.05, *p* = 0.071); however, there was a significant difference in the average daily intake (t = −3.42, *p* = 0.01), with females eating 2 ± 0.23 g (min–max: 1.74–2.28) and males eating 1.45 ± 0.19 g (min–max: 1.28–1.73) crickets/day. The total intake over the study by sex also showed a significant difference (t = −4.39, *p* = 0.002), with females eating 375 ± 43.72 g (min–max: 325–427) and males eating 258 ± 44 g (min–max: 198–323) crickets. Consequently, a significant difference was found in the final weight (t = −6.66, *p* < 0.001), with females weighing 2.86 ± 0.31 g (min–max: 2.36–3.09) and males weighing 1.91 ± 0.80 g (min–max: 1.79–1.98).

### 3.4. Skin Microbiome

**Figure 5:**
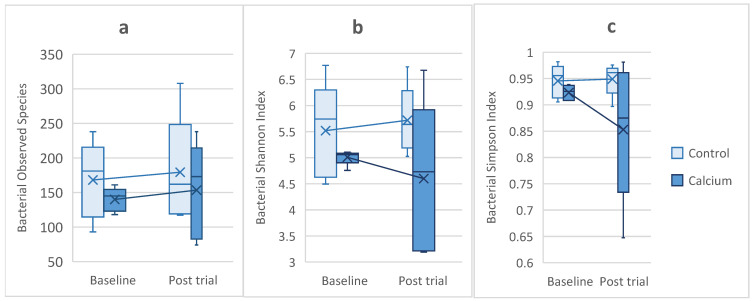

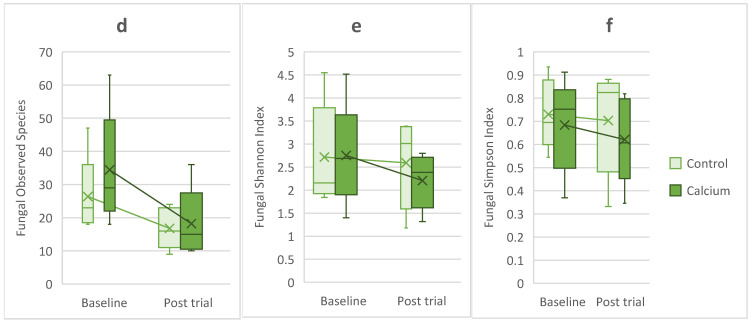
Alpha diversity indices (observed species, Shannon, and Simpson) of the bacteriome (**a**–**c**) and mycobiome (**d**–**f**) for the control and calcium groups at baseline and 180 days. No significant differences were found between group, time, sex, or their interaction (all *p* > 0.05). Boxes: 25–75th interquartile range; X: mean; horizontal line: median; whiskers: min–max.

**Figure 6:**
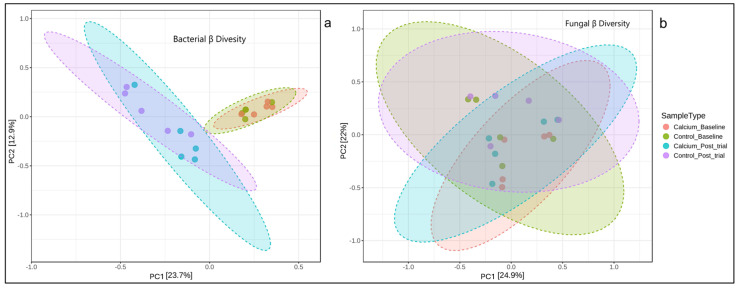
Principal coordinates analysis of beta diversity (β) for the bacteriome (**a**) and mycobiome (**b**) for control and calcium groups at baseline and 180 days. A significant difference in bacterial β diversity was found by sampling time (*p* = 0.001); however, no significant difference was found for fungal β diversity (F = 1.425, *p* = 0.098). Ellipses represent the 95% confidence intervals.

Calcium did not have a significant effect on the frogs’ bacterial or fungal skin microbiota. For the bacterial microbiota, no significant difference was observed in the alpha diversity indices (Observed ASVs, Shannon, and Simpson) among sex (all *p* ≥ 0.55), group (all *p* ≥ 0.389), sampling time (all *p* ≥ 0.451), or their interaction (all *p* ≥ 0.636) (Figure 5a–c). After the trial, the bacterial community of the control group had 179.4 ± 77.84 (min–max: 117–308) observed species, while the calcium group had 153.4 ± 69.20 (min–max: 74–238) (Figure 5a) observed species. These values were consistent with the baseline observed species for both groups, as the control group also had a higher observed species count at baseline (Figure 5a). The bacterial beta community diversity did show an overall significant difference between groups (F = 2.45, *p* = 0.001); however, the difference was only found by time (all *p* ≤ 0.01). The calcium and control groups were not significantly different at baseline (F = 0.939, *p* = 0.566) or 180 days (F = 1.408, *p* = 0.183) (Figure 6a).

There was also no significant difference in the alpha diversity indices for the fungal microbiota by sex (all *p* ≥ 0.085), group (all *p* ≥ 0.306), sampling time (all *p* ≥ 0.158), or their interaction (all *p* ≥ 0.202) (Figure 5d–f). At 180 days, the control group had 16.8 ± 6.22 (min–max: 9–24) observed fungal species, while the calcium group had 18.2 ± 10.57 (min–max: 10–36) observed fungal species (Figure 5d). Both groups showed a non-significant reduction from baseline, with a 36.4% decrease in the control group and 47.1% decrease in the calcium group (F = 2.24, *p* = 0.158). Fungal beta diversity was not significantly different between the groups (F = 1.425, *p* = 0.098) (Figure 6b).

**Figure 7:**
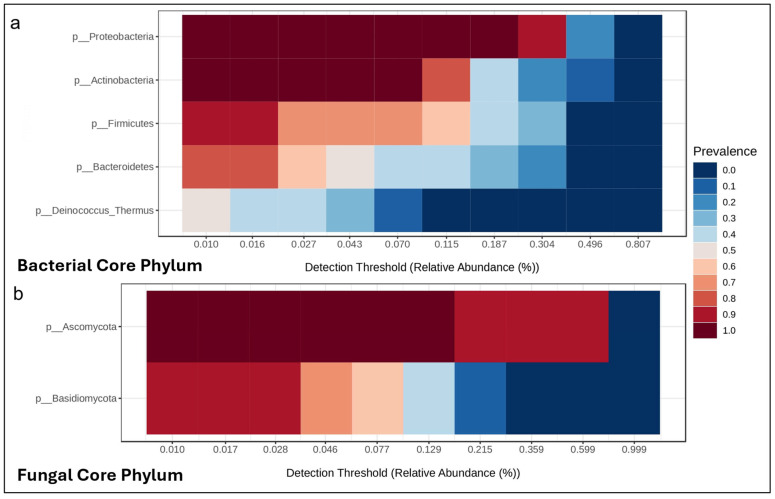
Heat map of the bacterial (**a**) and fungal (**b**) core phyla at 180 days, with a minimum relative abundance of 0.01% and sample prevalence of 20%.

The relative abundance of the taxonomic composition of the core phylum is represented in Figure 7. For the bacterial microbiota, the most abundant and prevalent phyla were Proteobacteria (42.22%), Actinobacteria (20.91%), Firmicutes (17.45%), Bacteriodetes (13.39%), and Deinococcus-Thermus (2.45%). Ascomycota (71.86%) and Basidiomycota (13.75%) were the most abundant fungal phyla.

### 3.5. Mortality

At 120 days of our study, one frog from the treatment group died. The histopathology revealed urothelial squamous metaplasia, cystitis, and urinary bladder rupture with septic coelomitis (File S1). While no other diagnostic test was performed, the histopathology findings suggested underlying urothelial squamous metaplasia. No evidence of NSHP was observed. A final mCT and microbiome sample could not be obtained on this animal.

## 4. Discussion

Based on the results of this study, enhancing a commercial cricket diet with calcium can be used to effectively transfer this essential mineral through gut-loaded crickets directly to subadult lemur tree frogs. This study is the first to demonstrate that a gut-loading period of only six hours with an 8% calcium (DM) diet is sufficient to effectively gut-load the crickets. While it is generally assumed this can be done, it is important to recognize that gut-loading crickets may not always directly serve to transfer the nutrients [25].

A cornmeal-based cricket diet with a low calcium concentration (1.28 ± 0.03%) was selected as the control diet because it represented a calcium concentration commonly found in cricket maintenance diets not intended for gut-loading purposes [16]. The control diet was modified to create the 8% calcium (DM) treatment diet based on the methodology described by Finke [22] using calcium carbonate, a calcium source recognized for having a higher rate of absorption and acceptance compared to other forms of calcium [16,18,21]. As expected, modifying the calcium concentration of the diet did not significantly affect the original moisture or DM content [22]. A small but statistically significant reduction in phosphorus concentration was also observed in the treatment diet. This difference reflects a dilutional effect associated with the increased calcium fraction of the diet, rather than the intentional manipulation of phosphorus content because no phosphorus source was added to the diet. Importantly, this difference did not result in significant differences in phosphorus concentrations in gut-loaded crickets.

As intended, a significant difference was found in the calcium concentrations between the two diets, with the treatment group calcium diet being 6.2 times higher in calcium compared to the control diet. While calcium requirements have not been established for frogs, including the lemur tree frog, the 8% calcium concentration provided to the treatment group was based on the calcium requirements established for other vertebrate species by the National Research Council (NRC) [40,41,42]. Moreover, a gut-loading diet with 8% calcium (DM) has been considered the standard for converting crickets from an inverse Ca:P ratio to a positive ratio for most herpetological species [16].

Traditionally, a 24 h gut-loading period has been considered the standard to increase the calcium concentration of house crickets [18,22,43]. The 6 h gut-loading period selected for this study was based on a previous study conducted by the authors, demonstrating that this shorter duration resulted in the highest calcium concentration and Ca:P ratio in house crickets [25]. Similarly to the cricket diets, calcium and Ca:P were the primary factors affected (increased) in the gut-loaded crickets. The crickets gut-loaded with the control diet did have calcium concentrations and Ca:P ratios below the recommended range for most vertebrate species [40,41,42] and similar to those reported for non-gut-loaded crickets (0.3% Ca with a 0.15:1 Ca:P ratio) [12,18,19,20], indicating limited calcium-carrying capacity in these crickets. In contrast, the 8% calcium treatment diet achieved a significantly higher calcium concentration (1.43 ± 0.44) and Ca:P ratio (1.23 ± 0.42) in the 6 h gut-loaded 2-week-old crickets, and these values were above the recommended values considered appropriate for ensuring adequate calcium intake in frogs [21,44]. There was a wider variation in the calcium concentrations and the Ca:P ratios for crickets from the treatment group compared to the control crickets. Because the treatment diet had to be prepared several times throughout the study period, homogenization to obtain the same exact percentage of calcium in diets was not possible. This has also been found to be a common problem with commercial diets prepared in large batches [23,45]. Moreover, there will always be some natural variation in the amount of calcium ingested in gut-loaded crickets because, unlike a dry kibble offered as a complete diet, individual food intake by the crickets and the timing for excretion of digesta—and thus, calcium—will vary. The palatability of these calcium-enriched diets can also influence the amount of diet consumed by the crickets [25]. These results should reinforce that nutrition studies are not an exact science and that relative and not absolute changes are important. Therefore, maintaining a consistent protocol for gut-loading insects is important to reduce variables that can alter cricket intake, such as temperature, humidity, fasting, and gut-loading time [21].

Throughout this study, frogs were offered a maximum of five gut-loaded crickets per day; however, on average, the animals ate from 2.5 to 4% of their body weight, with no significant differences noted between groups for the daily or total number of crickets consumed. There are limited studies measuring the amount of food captive frogs consume daily, and, to the authors’ knowledge, this study is the first to evaluate this in ex situ arboreal frogs. Because both groups gained weight and were in good body condition based on examination, the authors suggest that these daily consumption rates of 2.5–4% can be used as baseline measures for feeding captive subadult frogs under similar environmental conditions. Moreover, despite a higher calcium concentration in the treatment group, it did not translate into a substantial difference in body weight compared to the control group. This is a similar finding to previous studies in juvenile mountain chicken frogs (*Leptodactylus fallax*) [46] and oriental fire-bellied toads (*Bombina orientalis*) [47].

The lemur tree frogs started and ended the trial in the subadult developmental stage. At the end of this study, the frogs were approximately 13 months of age, which is less than the 18–24 months this species typically requires to achieve sexual maturity. In addition, the snout–vent lengths remained below the reported adult ranges for females (3.8–4.5 cm) and males (3.0–3.8 cm) [48,49]. These findings suggest that the frogs were still undergoing somatic development and should not have had extra calcium demands associated with reproduction. Moreover, the lemur tree frog’s growth rate is longer and slower in comparison with other species of anurans, such as *Xenopus tropicalis*, which can achieve sexual maturity within 5–6 months after metamorphosis [50]. There was a wide standard deviation noted in weight gain that was linked to sex. Sex could not be determined at the beginning of this study because the froglets were not sexually dimorphic; however, this changed by the end of this study. There was an unequal sex distribution between the groups, with more females in the treatment group compared to the control group. Because female lemur tree frogs are larger than males, with snout-vent lengths of 38–45 mm and 30–41 mm [49,50,51], respectively, the authors were not surprised by this result. Sex was also a limitation in another study with fire-bellied toads [47] and highlights the challenges of working with nondimorphic subadult species. Fortunately, there was no difference in bone density between the sexes. The authors believe that bone density was not affected because the frogs were subadults and not reproductively active. However, future studies evaluating the role of these diets on bone density in sexually mature animals are needed to determine if differences in diet impact reproduction.

The initial mCT scans showed no significant differences in TVOI HU, but subsequent scans revealed a significant increase over time. At 180 days, significant differences in TVOI HU were observed between the groups, indicating that crickets gut-loaded on an 8% calcium diet for six hours can transfer the mineral to lemur tree frogs. Previously, bone mineralization studies for anurans and reptiles used radiographic imaging and were unable to find a difference in bone density between groups. For example, Allen et al. [18] measured long bone radiographic opacities in mature fox geckos (*Hemidactylus garnotii*) and Cuban tree frogs (*Osteopilus septentrionalis*) after being fed crickets gut-loaded with 1 and 8% calcium diets for 6 weeks; however, no significant changes in radiographs were found in either species. In mammals, it has been estimated that 30 to 50% of bone must be depleted before conventional radiographic methods can detect the reduced density [51,52], so the time the geckos and frogs were followed, and their maturity, may have been limitations in detecting any changes. Galante-Mulki et al. [13] found significant radiographic changes in long bone structure and density in gliding leaf frogs (*Agalychnis spurrelli*) with metabolic bone disease after 13 to 15 months. In the present study, mCT was used for the lemur frogs because of an expected increased sensitivity over radiographs, especially because of the small size of the frogs. Our findings suggest that changes in bone density can be detected much earlier than previously described. Moreover, the significant differences in HU bone density measured between the two groups at 180 days were only 3.1%, a fraction of the 30–50% required for radiographs [52,53]. It is important to note that the lemur tree frogs used in this study were subadult frogs when the study started, while the radiographic studies primarily followed adult animals, where expectations for changes in bone density might not be expected to be as large [54]. Our findings suggest that mCT should be considered when assessing bone density in ex situ frog populations because it may help to detect changes in bone density earlier than radiographs or clinical signs, providing an early method of detection so that corrective methods in diet can be made.

Dual-energy X-ray absorptiometry (DEXA) was defined by the World Health Organization (WHO) Scientific Group on the Prevention and Management of Osteoporosis as the gold standard for measuring bone mineral density [55]. While DEXA has been used in some veterinary studies [56,57,58], its use has remained limited, and computed tomography is more commonly used to assess bone density, especially in exotic animals [17,59,60,61]. Patient size limited the value of DEXA in the present study. Previous studies found DEXA was not sensitive enough to measure bone density changes in guinea pigs (*Cavia porcellus*) [60] or rabbits (*Oryctolagus cuniculus*) [61], which are 200 to 600 times larger than the lemur frogs by weight. Instead, our study focused on measuring a TVOI from lemur tree frogs using mCT scans, along with ROI and BVOI. Lucas et al. [62] found that bone VOI was highly correlated and as sensitive as DEXA for a corresponding region of interest (ROI) in dog femurs. Although Shaw et al. [57] used VOI to measure bone volume, surface, thickness, and perimeter in New Zealand native frogs (*Leiopelma* sp.), to the authors’ knowledge, the current study is the first to evaluate mCT images in frogs, or any species, that not only used ROI or individual bones but also the whole skeleton to measure bone density. Interestingly, while ROI and BVOI measurements did not differ significantly between groups at 180 days, TVOI analyses showed significant differences, underscoring the importance of using whole-skeleton metrics for detecting subtle changes in bone density.

The presentation of NSHP cases can behave differently depending on multiple factors. For example, Vera et al. [63] found that mechanical loads experienced by frogs’ bones, depending on locomotion and habitat use, will adapt the bone structure to resist stress differently depending on species and, therefore, could be impacted differently when lacking the minimum requirements for healthy bone development. Galante-Mulki et al. [13] described radiographic changes in gliding leaf frogs that were mainly in long bones on radiographs, with histopathological changes also found in the *canalis vertebralis*, whereas King et al. [14] found radiographic changes in mountain chicken frogs in the long bones of the hind limbs and skulls. While most studies select an ROI from a specific section of the selected bone to measure bone density, in the present study, it was possible to take advantage of the small volume of the lemur tree frogs and adapt the software to select the whole-body skeleton to measure the HU mean bone density (TVOI). Therefore, using the skeleton total mean HU allowed the inclusion of any bone density reduction (expected from the control group), regardless of mechanical loads on the bones, and any high-calcium-concentration storage that could potentially exist (expected in the treatment group) in the paravertebral endolymphatic sacs [44]. This increased the sensitivity of the mCT scans because using only the femurs or vertebrae or an ROI of these bones would have missed the difference in HU between groups. Future studies should focus on how the individual bones of frogs respond to low-calcium diets to further refine the methods for measuring change in bone integrity.

Calcium digestibility played an important role in this study, especially for the control group. There was no correlation between total cricket intake and weight gain in the frogs, and no clinical or mCT signs of fibrous osteodystrophy suggestive of NSHP were observed in either group. Thus, regardless of total cricket or calcium intake, all the frogs absorbed sufficient calcium to grow at equal rates over the study period and develop sufficient bone health to prevent NSHP. Van Zijll Langhout et al. [60] suggested that mineral-depleted environments can influence amphibians to be highly efficient at digesting, absorbing, and storing minerals, and based on our findings, the authors agree. Moreover, Michaels et al. [46] found that frogs fed dusted crickets with daily calcium excreted twice as much calcium in their feces compared to those receiving supplementation only twice a week. We collected feces from the frogs to measure fecal calcium concentrations to further assess the digestibility of the calcium; however, the frogs produced insufficient sample weights (even when pooled) for measuring the calcium. Therefore, it is possible that frogs in the control group maximized their calcium absorption to ensure growth and bone development and that their absorption rate of calcium may have been higher than that of the treatment group. A recent study in blue-tongued skinks (*Tiliqua scincoides*) found that calcium absorption rates differed based on available calcium content, with digestibility being 1.7 times higher in the low-calcium (0.3%) group compared to the high-calcium (8%) group [64]. These findings should remind us that these animals have evolved to maximize their ability to absorb essential nutrients when availability is low and that creating diets with high concentrations of calcium may be unnecessary. This should be evaluated further because palatability can sometimes impact the acceptance of prey insects, especially when dusting the calcium onto the insects [24]. Our study did not assess the long-term effects (beyond 180 days) on the health, fitness, and calcium requirements in other life stages, such as reproduction, so these studies will be needed to further refine recommendations for ex situ populations of lemur tree frogs.

Environmental calcium is a potential source of this mineral for anurans and should be evaluated when taking a holistic approach to assessing calcium sources in an ex situ setting. It has been suggested that frogs can absorb calcium through their skin, so environmental sources of calcium (e.g., water) can help offset deficiencies from the diet, especially in animals on a low-calcium diet [14]. In the present study, frogs were all exposed to the same dechlorinated tap water, which had a calcium carbonate hardness range from 0 to 25 ppm. This value is considered a low to average amount of hardness and was not expected to have a significant effect on the frogs. This is further supported by the inability of the control frogs to acquire sufficient calcium from the environment to offset the differences noted in HU bone density between diets. However, this was not a defined hypothesis in our study and should be evaluated more rigorously to determine its value.

Allen et al. [18] have suggested that their study had no significant difference in bone measurements because of a lack of ultraviolet B (UVB) exposure. However, the authors of the present study disagree with this assumption and instead think the difference was more likely related to the low sensitivity of radiographs for measuring bone density and the short duration of the study. The authors did not use UVB in the present study on lemur tree frogs, but they did find significant differences in bone density between groups. Our study was initiated with subadult animals, utilized a more sensitive method to measure bone density, and was significantly longer in duration (180 days vs. 42 days). We specifically did not use UVB in this study to isolate our focus on the independent variable calcium. Adding UVB would have potentially altered this study by introducing a confounding variable. The sample size for the present study was already limited, so adding a UVB variable as an independent variable would have increased our risk for a type II error. All frogs could have been exposed to UVB as a baseline variable, but variable exposure rates by the frogs could have created an uncontrolled variable that could have impacted calcium absorption. The authors are currently assessing the impact of UVB on red-eye tree frogs (*Agalychnis callidryas*) in a separate study and have found the frogs to have different UVB exposure rates based on positioning within their enclosures (unpublished data, M.G.A). Van Zijll et al. [65] also found a significantly higher HU femur density between European common spadefoots (*Pelobates fuscus*) that received vitamin–mineral supplements without UVB exposure while housed indoors compared to those that did not receive oral supplementation but were exposed to natural UVB. Moreover, Antewis et al. [66] found that UVB exposure had no effect on the morphometrics or body condition of red-eyed tree frogs. Thus, the authors suggest that when evaluating frogs in ex situ settings, calcium supplementation, the duration of supplementation, and the type of diagnostic imaging are more important than UVB exposure for characterizing bone density. Having noted this, future studies evaluating the role of UVB under these conditions (e.g., developing animals, longer durations, and mCT) and oral vitamin D should be pursued to further elucidate the value and role of UVB for ex situ populations.

One frog from the treatment group died near the end of this study. The histopathology findings were more consistent with changes in hypovitaminosis A rather than NSHP [10,67]. Measuring vitamin A concentrations would have been required to confirm this suspicion; however, this was not possible. The animals did receive vitamin A supplementation in their diet, so it is uncertain if the squamous metaplasia was caused by hypovitaminosis A or was a response to a bacterial infection. No other frog in this study showed signs of hypovitaminosis A, and all remained clinically healthy through the trial, making it difficult to determine whether this individual, or any other, may have been in an early subclinical stage of hypovitaminosis A. Importantly, there was no evidence of NSHP or bone abnormalities, indicating that the mortality was not related to calcium intake and bone density outcomes.

Contrary to the effects observed with bone density, differences in dietary calcium did not affect the skin microbiome of lemur tree frogs. For the bacterial communities, the alpha diversity remained stable across groups, sexes, and sampling times, preserving its richness and evenness. It is important to highlight that the control group did have higher alpha diversity indices post-trial, but this pattern already existed at baseline, suggesting that values were related to natural individual variation rather than treatment effect. Similarly, beta diversity was not impacted by calcium. The only significant difference was observed by time, reflecting changes between baseline samples, which were collected well before the trial began, and the post-trial samples. These temporal shifts demonstrate the dynamic nature of the skin bacterial microbiota and its responsiveness to environmental conditions and natural fluctuations [68,69], rather than a direct impact of dietary calcium. In contrast, the mycobiota was more stable because neither the alpha nor beta diversity showed significant differences over time or between groups and sexes. This suggests that the fungal communities of these frogs may be more stable and less sensitive to short-term variations compared with bacterial communities; however, further studies are needed to explore this further. The taxonomic bacterial and fungal phyla identified at the conclusion of this study were consistent with those expected for anurans under human care [68,70]. Our study demonstrated that lemur tree frogs offered different dietary calcium diets for 180 days did not alter the diversity or composition of their skin microbiomes, suggesting that calcium at these concentrations and duration did not influence the skin microbiome like other nutrients, such as carotenoids [33], or that different concentrations or durations of calcium may be required.

There were several limitations in this study that should be addressed. First, the sample size was limited because of the availability of this species of frog and financial resources. Regardless, the primary hypothesis of concern, that differences in HU bone density would occur between dietary groups, was proven with the study sample size. Because we were able to prove the alternative hypothesis, the sample size was adequate to prevent a type II error. Sex was a factor that was difficult to randomly block into our study design because we started the study with subadult frogs. Ultrasound could possibly have assisted with sexing frogs, but at 1–1.5 g, we found it challenging. mCT with contrast may have also helped, but the frogs were too small to gain intravenous access. Fortunately, we were able to evaluate the role of sex as an independent variable in the final measurements, and it did not influence bone mineralization; we suspect this was because the frogs were not reproductively active. None of the frogs developed signs of NSHP over the course of this study based on mCT findings. Because the lemur tree frogs were <3 g at the conclusion of this study, plasma biochemistry testing to evaluate calcium and phosphorus concentrations was not possible. However, since early-stage and subclinical NSHP are usually detectable by radiography [17] and reptiles and amphibians with SNHPNSHP are often normocalcemic and normophosphatemic, we do not expect the biochemistry testing would have offered much [44,65]. We did have a four-month period between acquiring the frogs and starting the project due to logistics and the need to complete a sedation protocol for the mCT [37]. Being able to start the project earlier may have shown a larger difference between groups because the frogs were younger and in a growth phase. However, studying the frogs at the time we did during later growth and prior to reproduction and finding the difference in HU between groups reinforces that skeletal changes can occur during these later stages of growth too. Future temporal studies should evaluate how these different life stages are impacted by the frogs’ nutrition. Finally, skin microbiome samples were collected only at the end of this study due to financial limitations. Although compared with previously collected baseline data, the lack of pretrial sampling does not allow direct assessment of how time alone influenced bacteriome changes over the trial.

## 5. Conclusions

This study provides practical guidance for the care of subadult lemur tree frogs under ex situ settings. Crickets gut-loaded with 8% calcium (DM) diet for 6 h were sufficient to transfer calcium and increase bone density, as measured by HU, over 180 days. Frogs fed crickets gut-loaded with a 1.3% calcium (DM) did not develop NSHP, but their bone density was lower than in the 8% calcium group. Moreover, assessing bone density through TVOI HU of mCT scans was more sensitive for detecting differences in bone density than the selected ROI and BVOI. Additionally, this study found that subadult lemur tree frogs consume 2.5 to 4% of their body weight daily. Finally, skin microbiome structure was driven more by natural temporal variation than by calcium concentration in the diet.

## Figures and Tables

**Table 1:** Descriptive statistics for the calcium, phosphorus, dry matter, and moisture contents of the two gut-loading diets used in this study. Calcium (*p* < 0.001) was significantly higher in the treatment group compared with the control group. Phosphorus (*p* < 0.001) was significantly lower in the treatment group compared with the control group.

	Calcium	Phosphorus	Dry Matter	Moisture
**Control group** **(1.3% calcium)**	1.28 ± 0.03 (min–max: 1.26–1.31)	0.9 ± 0.03 (min–max: 0.87–0.92)	88.6 ± 0.6 (min–max: 87.9–89.1)	11.4 ± 0.6 (min–max: 10.9–12.1)
**Treatment group** **(8% calcium)**	7.9 ± 0.75 (min–max: 6.59–8.78)	0.79 ± 0.02 (min–max: 0.76–0.81)	89.56 ± 1.14 (min–max: 87.8–90.4)	10.45 ± 1.14 (min–max: 9.6–12.2)

All values were analyzed as percentages based on DM.

**Table 2:** Descriptive statistics for calcium, phosphorus, dry matter, and moisture contents of cricket samples from the treatment and control groups. Calcium (*p* = 0.005) and Ca:P ratio (*p* = 0.007) were significantly higher in the treatment group compared with the control group.

	Calcium	Phosphorus	DM	Moisture	Ca:P Ratio
**Control group (1.3% calcium)**	0.16 ± 0.017 (min–max: 0.14–0.18)	1.17 ± 0.03 (min–max: 1.13–1.19)	26 ± 2.21 (min–max: 23.6–28.5)	74 ± 2.21 (min–max: 71.5–76.4)	0.14 ± 0.02 (min–max: 0.12–0.16)
**Treatment group (8% calcium)**	1.43 ± 0.44 (min–max: 0.97–1.87)	1.17 ± 0.05 (min–max: 1.10–1.23)	25.43 ± 1.68 (min–max: 23.1–27)	74.56 ± 1.68 (min–max: 73–76.9)	1.23 ± 0.42 (min–max: 0.82–1.7)

All values were analyzed as percentages based on DM.

**Table 3:** Lemur tree frog bone density (mean ± SD) measured in HU on mCT scans of select volumes and regions of interest from the right and left femurs and third vertebra 180 days after initiating this study. No significant difference was found between groups.

Segment	Bone Density (HU)	t	*p*
Right femur VOI	617.97 ± 33.70 (min–max: 576.52–686.47)	0.790	0.452
Right femur ROI	1156.52 ± 108.91 (min–max: 885.74–1256.19)	1.12	0.295
Left femur VOI	621.69 ± 32.37 (min–max: 569.55–667.40)	0.966	0.362
Left femur ROI	1246.03 ± 144.58 (min–max: 1003.15–1404.08)	0.270	0.794
Third vertebra VOI	712.04 ± 37.74 (min–max: 668.94–774.23)	0.891	0.399
Third vertebra ROI	784.24 ± 45.29 (min–max: 706.2–879.44)	0.451	0.664

## Data Availability

The original datasets presented in this study are included in this article; further inquiries can be directed to the corresponding author.

## Supplementary Materials

The following supporting information can be downloaded at https://www.mdpi.com/article/10.3390/ani16040660/s1. File S1: Histopathology analysis of the frog in the treatment group that died.

## Abbreviations

The following abbreviations are used in this manuscript:

SNHP	Secondary nutritional hyperparathyroidism
Ca:P	Calcium-to-phosphorus ratio
DM	Dry matter basis
HU	Hounsfield units
mCT	Micro-computed tomography scan
ROI	Region of interest
BVOI	Bone volume of interest
NRC	National Research Council
UVB	Ultraviolet B
DEXA	Dual-energy X-ray absorptiometry
WHO	World Health Organization
