# Supplementary material for: Effects of Two Different Dietary Calcium Concentrations on Bone Density and Skin Microbiome in Lemur Tree Frogs (Agalychnis lemur)
Source: Animals (Basel). 2026 Feb 19;16(4):660. doi: 10.3390/ani16040660 (PMC12937340; doi:10.3390/ani16040660)
Supplement: Supplementary file 1 [file animals-16-00660-s001.zip › animals-4117550-supplementary.pdf]

Louisiana Animal Disease Diagnostic Laboratory
LA
Phone: (225) 578-9777 Fax: (225) 578-9784

Report Date: 8/4/2023

Final Report

Date Received: 7/31/2023 **Case Coordinator:** Naomi Falconnier **Accession No:** L2312097
VTH #: 258767

LSU
DR. M. GRACIELA AGUILAR
LA

Email: magui32@lsu.edu

Associated Parties

Clinician	LSU Dr. M. Graciela Aguilar	LA
Owner	Fluker Farms Zoo Med Support F BG006846	
LSU Internal Vet Practice	LSU/SVM (VCS)	Veterinary Medicine Bldg<br>Baton Rouge, LA 70804<br>(225) 578-9553
LSU Internal Vet Practice	LSU/SVM Small Animal Clinic	
LSU Internal Veterinarian	LSU Dr. Mark Mitchell	LA

Reference Data

PG #: 2136 Supervising Pathologist: E. Sasaki

Animal Information

Name	Taxonomy	Sex	Age
LF1	Anura - Frog	Male	8 Months

Diagnosis

Urothelial squamous metaplasia
Cystitis and urinary bladder rupture with septic coelomitis

Morphologic Diagnoses:

Urinary bladder:
-- Squamous metaplasia, widespread, moderate to marked
-- Cystitis, histiocytic, multifocal, minimal to mild, acute to subacute with focal rupture and intralesional and intra-histiocytic short bacilli
Coelom: Septic coelomitis, fibrinous and histiocytic, widespread, moderate, acute to subacute with numerous extracellular and intra-histiocytic short bacilli, segmental intestinal serositis and leiomyositis, and pericarditis
Lung, liver, spleen: Circulating intra-monocytic short bacilli, numerous

Clinical Data

Research animal. Current in the treatment group of a nutritional study. Healthy animal, eating very well and with no clinical signs until this morning. It was lethargic, bloated and without eating crickets Saturday night/Sunday morning. It was expose to extreme temperatures since Thursday, 67.6 to 81F.

Gross Findings

Submitted is one small container, labeled “FROG #1, CWR frog”, which contains 1 whole frog that measures 3 cm long, from the tip of the rostrum to the anus. The whole frog was decalcified, and serial sections of the head are placed in cassette A, and serial sections of the body are placed in cassettes B and C. A representative section of the left pelvic limb is also placed in cassette C.

Histopathology

Urinary bladder: A tubular organ within the caudal coelomic cavity is largely lined by a keratinizing squamous epithelium with a few small areas of urothelial lining and consists of a musculature wall that is characterized by small discontinuously arranged bundles of smooth muscle cells (consistent with urinary bladder). Accumulating within the lumen is sloughed keratin with low numbers of interspersed foamy macrophages and many colonies of short bacilli. The wall is regionally necrotic and ruptured, with loss of differential staining and fragmentation, and is heavily colonized by similar bacteria. Along the perforated margins, the urothelial lining is sloughing and hypereosinophilic, and urothelial cells are individualized and are degenerate to necrotic.

Serosal surfaces (coelom, pericardial sac, hepatic capsule, intestines): Adjacent to the focally ruptured urinary bladder and multifocally scattered throughout the coelomic cavity and pericardium are many fibrin aggregates which entrap macrophages with intracytoplasmic bacteria as well as extracellular bacteria and hemorrhage. Lining mesothelial cells are multifocally mild to moderately hypertrophied, and multifocally colonizing the coelomic serosal surfaces are numerous similar extracellular and intrahistiocytic bacteria.

Small intestines: In one examined section, the lamina propria is expanded by hemorrhage and infiltrated by low numbers of macrophages. Intravascular monocytes and histiocytes within the lamina propria contain numerous rod-shaped bacteria which are also extracellular and heavily colonizing the muscularis and serosa. These changes are not associated with any alteration in the overlying mucosa.

Spleen, liver: Many similar extracellular and intracellular short bacilli are within circulating monocytes and resident splenic macrophages.

Lung: Circulating within multiple blood vessels are similar bacteria which are multifocally contained within monocytes. A few foamy macrophages are closely associated with the faveolar lumen which also contain small amounts of proteinaceous material colonized by similar bacteria.

Brain, spinal cord, eyes, ear (internal, middle), oronasal cavity, trachea, esophagus, stomach, colon, cloaca, anus, kidneys, adrenal glands, testicles, muscle, skin, bone, bone marrow: No significant microscopic alterations are noted in the examined sections.

Comments

The main microscopic findings of significance include urinary bladder squamous metaplasia, minimal to mild bacterial cystitis, and focal urinary bladder rupture with bacterial coelomitis and sepsis. While the exact cause of urinary bladder rupture in this frog is uncertain, retrograde bacterial cystitis followed by urinary bladder rupture is speculated to have stemmed from the underlying urothelial squamous metaplasia.

Client Report History

Report Type	Delivery Method	Sent To	Date Sent
Preliminary	Email	magui32@lsu.edu	7/31/2023 12:36 PM
Final	Email	magui32@lsu.edu	8/4/2023 11:37 AM
